# Circulating Cell-Free DNA as a Prognostic and Molecular Marker for Patients with Brain Tumors under Perillyl Alcohol-Based Therapy

**DOI:** 10.3390/ijms19061610

**Published:** 2018-05-30

**Authors:** Giselle Faria, Emanuelle Silva, Clovis Da Fonseca, Thereza Quirico-Santos

**Affiliations:** 1 Department of Cellular and Molecular Biology, Institute of Biology, Fluminense Federal University, Niteroi, Rio de Janeiro 24020-141, Brazil; gisellefaria@id.uff.br (G.F.); sraferreira.bio@hotmail.com (E.S.); 2 Department of Specialized Medicine, School of Medicine, Fluminense Federal University, Niteroi, Rio de Janeiro 24020-141, Brazil; clovis.orlando@uol.com.br

**Keywords:** cell-free DNA, circulating nucleic acids (CNA), perillyl alcohol, intranasal therapy, glioblastoma, brain metastasis, magnetic resonance image (MRI)

## Abstract

Tumor infiltration into brain tissue usually remains undetected even by high-resolution imaging. Molecular markers are used to increase diagnostic accuracy, but with limited continuous monitoring application. We evaluated the potential of circulating cell-free DNA (cfDNA) as a molecular indicator of the response to therapy by the intranasal administration (ITN) of perillyl alcohol (POH) in brain tumors. The cohort included 130 healthy subjects arranged as control-paired groups and patients at terminal stages with glioblastoma (GBM, *n* = 122) or brain metastasis (BM, *n* = 55) from stage IV adenocarcinomas. Serum cfDNA was isolated and quantified by fluorimetry. Compared with the controls (40 ng/mL), patients with brain tumors before ITN-POH treatment had increased (*p* < 0.0001) cfDNA median levels: GBM (286 ng/mL) and BM (588 ng/mL). ITN-POH treatment was significantly correlated (rho = −0.225; *p* = 0.024) with survival of >6 months at a concentration of 599 ± 221 ng/mL and of <6 months at 1626 ± 505 ng/mL, but a sharp and abrupt increase of cfDNA and tumor recurrence occurred after ITN-POH discontinuation. Patients under continuous ITN-POH treatment and checked with brain magnetic resonance imaging (MRI) compatible with complete response had cfDNA levels similar to the controls. cfDNA may be used as a noninvasive prognostic and molecular marker for POH-based therapy in brain tumors and as an accurate screening tool for the early detection of tumor progression.

## 1. Introduction

Glioblastoma (GBM) and brain metastases (BM) disseminate into the brain parenchyma due to their highly proliferative, anabolic and invasive properties and as a result of their interactions with cerebral microvascular endothelial cells that attenuate metabolic pressures and increase the probability of tumor dissemination [1]. Complete surgical tumor resection is difficult considering the invasive, diffuse and ill-defined borders in the brain parenchyma favoring tumor recurrence [2,3]. Despite refinements in the efficiency of brain imaging techniques for assessing anatomic and structural features, small tumor niches, critical for early tumor detection and/or recurrence, may not be detectable [1,4]. Other neuroimaging techniques, such as positron emission tomography (PET) providing data regarding tumor metabolism [4,5], however, also face constraints due to the inability to distinguish radionecrosis from inflammatory infiltrate and tumor activity areas [6,7,8]. Although molecular markers increase histological diagnosis accuracy [9], there is also the need to consider the dynamic and heterogeneous nature of the tumor mass characterized by marked genomic instability, somatic mutations and epigenetic changes resulting from cytotoxic chemotherapy and ionizing radiation [10,11,12]. Therefore, due to the limited therapeutic options for brain tumors resulting in inexorable recurrence and drug resistance, there is an urgent need for new therapeutic alternatives to increase the patients’ quality of life when they still face a dismal prognosis.

Circulating nucleic acids (CNA) have long been detected in human serum or plasma as a result of biological processes such as apoptosis, necrosis or active release from living cells [13]. High CNA levels are often present in several medical conditions including inflammation [14], but low molecular weight double stranded fragments ranging from 21 kb to less than 0.5 kb are often detected in plasma from cancer patients [15,16,17]. Due to the advantages of liquid biopsy, cell-free DNA (cfDNA) has potential clinical applications for cancer patients since it is a representative biological source for identifying tumor genetic and epigenetic alterations. Cell-free DNA may be especially suitable for cases in which complete surgical resection is impossible, since the small DNA fragments often have similar molecular alterations identified by tumor biopsy [17,18]. Moreover, quantification of cfDNA can be a potential biomarker for accurately discriminating cancer patients from healthy individuals [19].

Perillyl alcohol (POH) is a naturally occurring monoterpene, previously investigated via intranasal administration under a Phase II clinical trial, with very promising results for treatment of recurrent GBM temozolomide (TMZ)-resistant patients [20,21]. The therapeutic activity of POH is due to its cytotoxic, pro-apoptotic, anti-inflammatory and antiangiogenic properties [22], with no adverse effects reported so far for the intranasal (ITN) route of administration [20]. The blood–brain barrier (BBB) is compromised in patients with malignant brain lesions, but tumor progression may remain asymptomatic until patients are severely ill, partly because cerebrospinal fluid collected as an invasive procedure may not be routinely used for monitoring patients’ therapy response. In this scenario, the present study aimed to test the hypothesis that circulating cfDNA as a representative molecular marker may be utilized for disease monitoring of patients with brain tumors under POH-based therapy.

## 2. Results

### 2.1. Detection of Circulating cfDNA in Serum of Patients with Brain Tumors

**Figure 1:**
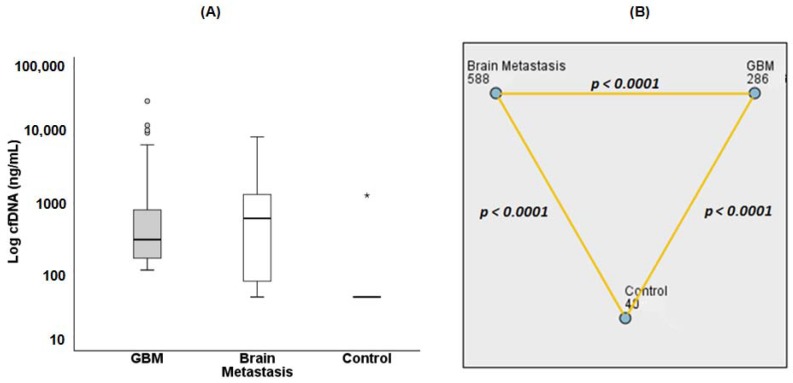
Median serum levels of circulating cell-free DNA (cfDNA) in the study groups. (**A**) Box plot graph of cfDNA levels in patients with glioblastoma (GBM) (*n* = 122, median = 286 ng/mL), brain metastasis (*n* = 55; median = 588 ng/mL) from adenocarcinoma stage IV (breast *n* = 19; lung *n* = 19; pancreas *n* = 2; colon *n* = 11; prostate *n* = 4) before intranasally administrated perillyl alcohol (ITN-POH) therapy compared with healthy control subjects (*n* = 130, median = 40 ng/mL). (**B**) SPSS statistical package graphics output of median cfDNA pairwise comparisons of groups including the outliers, with respective significant differences (* *p* < 0.0001).

Cerebrospinal fluid (CSF) has been described as the most appropriate biological sample for assessing cfDNA in brain tumors [23]; however, its collection is an invasive procedure that cannot be routinely used for monitoring responses to therapy. Therefore, the viability for determining cfDNA levels from freshly processed serum of patients with GBM and brain metastasis was tested (Figure 1). The blood samples were quickly processed since circulating cfDNA has a half-life of less than 2 h [24].

Comparing the mean values of cfDNA measurements (Table S1), healthy subjects consistently had very low levels of serum cfDNA (mean = 40.48 ± 0.52 ng/mL) in contrast to significant (*p* < 0.0001) increases of cfDNA in serum from patients with GBM (mean = 1237.50 ± 314.29 ng/mL) and brain metastasis (mean = 1237.44 ± 255.22 ng/mL). These results were obtained before ITN-POH treatment (Figure 1). Interestingly, both groups with intracranial tumors showed similar mean levels of circulating cfDNA and approximately a 30-fold increase compared to the control group. Since there were very wide distributions of the data sets and the mean statistical evaluations were greatly influenced by extreme values (outliers), a median pairwise comparison of these groups was performed. Significant differences between the median values of each group were obtained (*p* < 0.0001, Figure 1B and Table S1). The results suggest that cfDNA may be a strong molecular candidate capable to differentiating healthy subjects from cancer patients with brain tumors.

### 2.2. Diagnostic Power of cfDNA Serum Levels for Brain Tumor Evaluation

**Figure 2:**
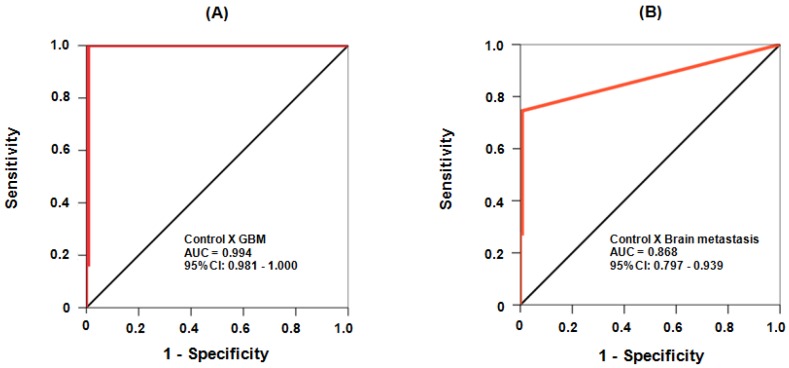
Receiver operating characteristic (ROC) analysis of sensitivity and specificity of cfDNA serum levels for distinguishing healthy control from those with GBM (**A**) and brain metastasis (**B**). The black diagonal line defines a test with no discriminative power (it is, random chance result with zero diagnostic capability, equivalent to an area under the curve (AUC) = 0.5); the red line represents accuracy and increasing diagnostic power the more distant its plot is located from the diagonal black line.

We further evaluated the sensitivity and specificity of cfDNA for discriminating a healthy control from those with GBM and brain metastasis. For this study, a receiver operating characteristic (ROC) curve statistical analysis was selected to evaluate the diagnostic power of cfDNA. Areas under the curves (AUC) were considered critical for establishing the accuracy of cfDNA as a molecular entity [24]. Both ROC plots (GBM and brain metastasis) were very close to the left-hand and the top borders of the ROC space (Figure 2); an indication that the test had a high degree of accuracy. In addition, the observed AUC value of 0.994 confers to cfDNA an excellent capacity for differentiating GBM from healthy subjects (Figure 2A). Moreover, the AUC value of 0.868 for brain metastasis (Figure 2B) also indicates cfDNA as a suitable test for distinguishing healthy subjects from patients with brain metastasis (Figure 2B).

### 2.3. Influence of POH-Based Therapy upon cfDNA Levels and Patient Survival

**Figure 3:**
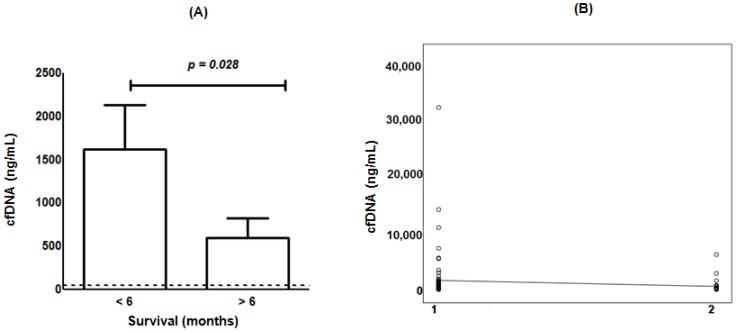
Circulating cfDNA level and GBM survival during ITN-POH therapy (mean values ± standard error). (**A**) cfDNA levels for GBM survivors of less than 6 months (1626 ± 505 ng/mL) and more than 6 months (599 ± 221 ng/mL). Dotted line indicates the control cfDNA level. (**B**) Respective correlation 1: <6 months; 2: >6 months; rho = −0.225; *p* = 0.024).

Cell-free DNA is mainly released by proliferating tumor cells undergoing necrosis and/or apoptosis [17]. Considering that POH presents pro-apoptotic properties [20], it was relevant to verify a possible relationship between cfDNA levels and the survival of patients with brain tumors (GBM). In this context, a period of 6 months (24 weeks) was considered as a cut-off value for assessing enhanced survival. The results showed (Figure 3A) a significant difference (*p* = 0.028) between the mean cfDNA serum levels of patients who survived more than 6 months (599 ± 221 ng/mL) compared with those that survived less than 6 months (1625.83 ± 505 ng/mL, *p* = 0.028), with a 2.7-fold difference between the groups. The correlation test between patients’ survival at 6 months and cfDNA serum levels indicates (Figure 3B) a weak, significant and negative correlation between variables (rho = −0.225; *p* = 0.024; *n* = 122). Therefore, cfDNA may be regarded as a candidate molecular biomarker for prognostic evaluation of patients with brain tumors under POH-based therapy.

### 2.4. Effect of POH-Based Therapy on cfDNA Levels and the Disease Process

**Figure 4:**
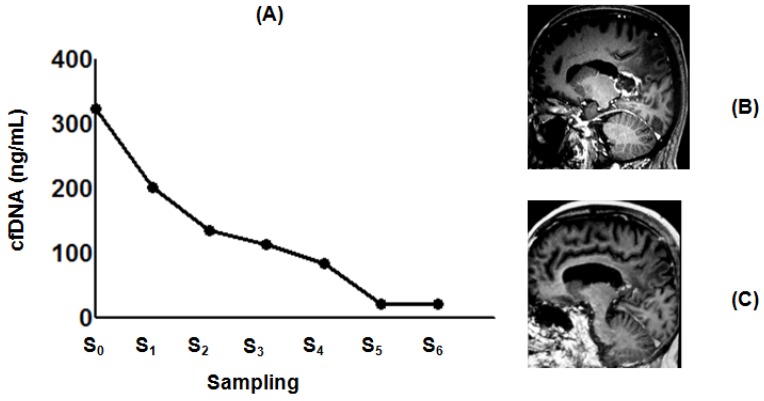
Figure of a GBM patient with a complete response to ITN-POH treatment. MRI at the time of protocol inclusion (**B**) corresponded to high cfDNA levels (**A**, S_0_ = 323 ng/mL, February 2008). ITN-POH therapy caused a consistent sharp reduction of cfDNA serum levels (**A**, S_1_ = 201 ng/mL, March 2009; S_2_ = 135 ng/mL, June 2010; S_3_ = 113 ng/mL, September 2011; S_4_ = 85 ng/mL, March 2012). Continuous ITN-POH therapy caused a 16-fold reduction of cfDNA levels after 6 years. (**A**, S_5_ = 20 ng/mL, February 2013; S_6_ = 20 ng/mL, July 2014) which was compatible with no detectable brain MRI lesion (**C**).

**Figure 5:**
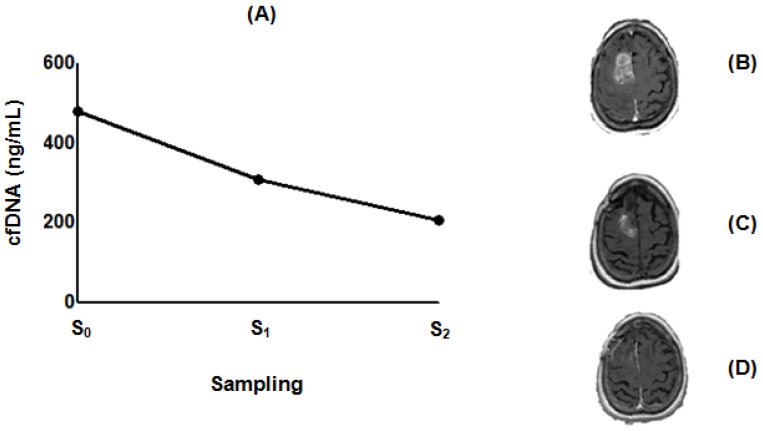
Figure of a GBM patient with a good response to ITN-POH based therapy. (**A**) cfDNA levels before and during treatment. Remarkable tumor mass identified by MRI at the time of protocol inclusion (**B**) and before ITN-POH therapy was accompanied by a high level of cfDNA (**A**, S_0_ = 479 ng/mL; October 2005). Therapy resulted in impressive tumor mass regression (**C**) and a marked decrease of cfDNA (**A**, S_1_ = 308 ng/mL; April 2007). MRI compatible with stable disease was observed after 4 years of ITN-POH therapy (**D**) and a two-fold decrease compared to initial cfDNA measurement (**A**, S_2_ = 205 ng/mL; December 2009).

**Figure 6:**
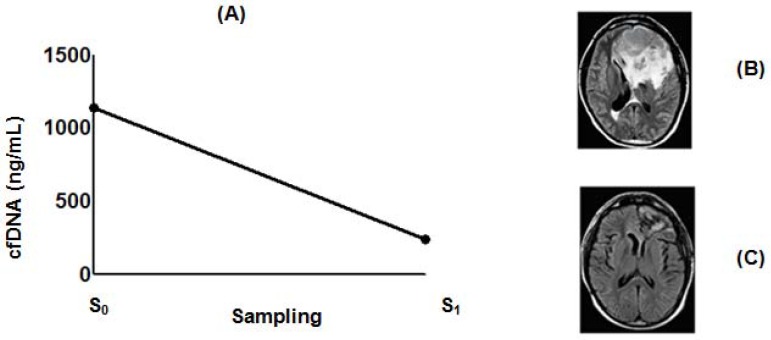
Figure of a patient with brain metastasis from prostate adenocarcinoma IV under ITN-POH based therapy. Initial brain image (**B**) corresponding to high levels of circulating cfDNA at the time of ITN-POH protocol inclusion (**A**, S_0_ = 1140 ng/mL; June 2006). ITN-POH therapy caused a five-fold decrease of circulating cfDNA (**A**, S_1_ = 234 ng/mL; December 2006) with MRI pattern compatible with stable disease (**C**).

**Figure 7:**
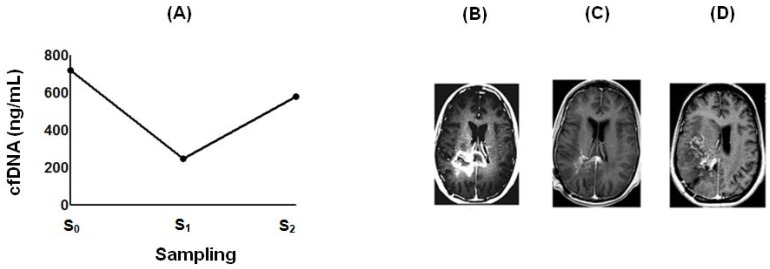
Figure depicts the effect of interrupting ITN-POH therapy. Intense signal with brain MRI (**B**) at the time of a GBM patient protocol enrollment correlated with high serum levels of circulating cfDNA (**A**, S_0_ = 720 ng/mL, November 2009). The therapy resulted in a three-fold decrease of cfDNA levels (**A**, S_1_ = 247 ng/mL, November 2012) and marked tumor mass reduction (**C**), but images of tumor recurrence (**D**) and a sharp increase of cfDNA serum levels (**A**, S_2_ = 580 ng/mL; February 2013) were simultaneously identified after 3 months of therapy discontinuation.

Since cfDNA level is considered a consistent indicator of tumor dynamics [17], it was important to verify whether quantitative changes in circulating cfDNA would correlate with the disease process and response to POH-based therapy. For this, sequential analyses of cfDNA from serum samples of patients with GBM and brain metastasis were performed. Results showed that POH-based therapy reduced cfDNA levels (Figure 4, Figure 5, Figure 6 and Figure 7). In the GBM patients, a decrease of circulating cfDNA levels occurred from 2.3 to 16-fold, reaching serum levels compatible with the normal physiological range. In addition, a brain MRI pattern equivalent to a complete response to POH-based therapy (Figure 4 and Figure 5) and longer survival (higher than 6 years) was observed. Regarding brain metastasis, POH-based therapy induced approximately a five-fold decrease of cfDNA concentration (Figure 6) after 6 months of treatment, with MRI showing huge tumor mass regression and long survival (>6 months). The meaningful effect of POH-based therapy is reinforced by GBM recurrence after interruption of ITN-POH treatment (Figure 7). A patient with stable disease after 3 years under continuous ITN-POH treatment had approximately a three-fold decrease of cfDNA levels (Figure 7A) and marked reduction of tumor size, as verified by brain MRI image exams (Figure 7B,C). However, 3 months after discontinuation of treatment, a two-fold increase on circulating cfDNA serum levels (Figure 7A), and a brain image compatible with tumor recurrence and progressive disease (Figure 7D) occurred. In conclusion, cfDNA emerges as a molecular marker for assessing responses to POH-based therapy and its serum levels can reflect the dynamics of malignant brain tumor biology.

## 3. Discussion

The current study showed that patients with brain tumors (brain metastasis and GBM) present higher concentrations of circulating cfDNA corresponding to a 30-fold increase compared with healthy individuals. Such results confirm cfDNA as a valid and accurate molecular indicator for distinguishing healthy patients from those with brain tumors. Moreover, cfDNA levels further reflect disease status and are an important tool for disease monitoring of patients with brain tumors under ITN-POH therapy.

Circulating cfDNA fragments released mostly by proliferating or dying tumor cells undergoing apoptosis and/or necrosis are digested by hydrolyzing enzymes (e.g., DNases) in the blood and circulate preferentially as mono and oligonucleosomes [24]. High concentrations of cfDNA in cancer patients may be due to low DNase levels and/or high DNase inhibitors [25], although distribution of cfDNA in the blood of cancer patients also depends on tumor localization [26]. Hence, invasive tumors that outgrow their blood supply contain larger regions of necrosis and upregulated protease activities that correlate with distant metastasis [24]. Primary brain tumors hardly ever produce metastasis at distant organs despite being highly infiltrative, whereas brain metastasis is often influenced by variables such as primary tumor site, the number and location of metastasis and by complex interactions within different brain microenvironments [27]. In the present paper, although the cohort of terminal patients (*n* = 55) with brain metastasis was relatively small and had a heterogeneous distribution concerning the primary tumor location (breast, lung, pancreas, colon, and prostate), however, circulating cfDNA values were closely associated with the response to ITN-POH therapy. In addition, the great variability in circulating cfDNA concentrations observed among patients with brain metastasis (40 to 9330 ng/mL), and among GBM patients (101 to 31,350 ng/mL), as well as the significant difference between serum cfDNA median values (GBM = 588 ng/mL; BM = 286 ng/mL, *p* < 0.0001) may be related to distinct tumor biology and location of tumor lesions within the brain parenchyma.

It is also important to consider any methodological differences between the extraction and quantification of circulating cfDNA, as well as the type of tumor examined and any therapeutic intervention [28,29,30]. However, the present data are in agreement with previous reports showing increased cfDNA serum levels in patients with epithelial malignancies and metastasis in comparison with healthy controls [31,32,33,34]. A very important finding in the present study was the viability of detecting circulating cfDNA in freshly processed serum of patients with metastatic brain tumors and recurrent GBM. This is in contrast with previous reports that recommended CSF as the biological sample for cfDNA evaluation of intracranial tumors, including gliomas [23,35]. This divergence may partially be related to the blood–brain barrier integrity that is still present at early stages of malignant transformation, but disruption is currently observed during progression and tumor recurrence [36]. Also, in the present study, the cohort was composed exclusively of recurrent GBM patients under palliative care, in contrast to Shi et al. that included mostly patients with low grade gliomas (grade I to III) and only a few (19%) GBM (grade IV) according to the WHO histological classification [35].

cfDNA is also suggested as an indicator of tumor progression, since high levels are associated with late stages of disease and metastasis in contrast to localized disease [23,37]. The present results also verified by sequential monitoring of individual patients confirm cfDNA serum levels as a good indicator of the response to POH therapy, and that this is directly correlated with brain MRI images. Since it is necessary to have 5 × 10^7^ cells to produce detectable amounts of cfDNA, but 10^9^ cells are required for image capture by high-resolution computed tomography [38,39], cfDNA emerges as a strong candidate for early indication of tumor recurrence. This would help in choosing the appropriate intervention, for example, in cases of non-responsive patients, avoiding ineffective therapies as well as undesirable side effects and related costs. The prognostic properties attributed to circulating cfDNA are also relevant for stroke patients with negative neuroimaging within 24 h of onset of symptoms, also reinforcing its value as a simple and non-invasive molecular indicator for early detection of a neuropathological process [40].

GBM is considered a rare disease at the Orphanet database (ORPHA: 360 glioblastoma http://www.orpha.net/consor/cgi-bin/Disease_Search_Simple.php?lng=EN) and is currently listed at WHO ICD-10 version 2016 (http://www.icd10data.com/ICD10CM/Index/G/Glioblastoma). In this regard, this study solely included terminal patients under palliative care. Unfortunately, therefore, it was not possible to reach a strict time-point for periodic sampling during ITN-POH treatment, although continuous monitoring had been performed. Despite this, the results provided strong evidence for the value of cfDNA monitoring for patients with brain tumor. Although cfDNA has not yet been established as oncologic clinical routine practice, elevated levels of circulating cfDNA in the blood of patients with cancer have been shown to reflect the characteristics of tumor malignancy, and to harbor genetic and epigenetic alterations at high risk for relapse [24]. Hence, clinical establishment of cfDNA as molecular marker should combine the quantitative determination with screening of specific molecular alterations such as IDH mutations and MGMT methylation status.

## 4. Materials and Methods

This retrospective study was carried-out with patients attending the out-patient Neurosurgical Unit in the Antonio Pedro University Hospital, Niteroi, Rio de Janeiro, Brazil and enrolled on Phase I/II clinical trials to assess the efficacy of intranasal administration of the monoterpene perillyl alcohol, POH. This project was approved by Ethics Committee of the University Hospital Medical School (HUAP-UFF) and Brazilian Ministry of Health (CONEP 9681 number 25000.009267/2004-25; approved on 12 July 2004), and is also in compliance with principles laid down in the Declaration of Helsinki. Each patient and the next-of-kin provided the informed consent before the protocol was executed.

### 4.1. Study Population

Inclusion criteria at enrollment in this study required adult subjects with brain tumors under palliative care and under symptomatic treatment after failing to respond to previous standard treatment, including surgery and/or radiation and multimodal chemotherapy specific for brain tumors. No patient was under radiation therapy or chemotherapy at the moment of inclusion in the study and any such treatment was already completed more than 4 weeks previously. The cohort, established to assess therapeutic efficacy of intranasal administration of POH, was composed of 122 patients with 74 males (60.7%) and 48 females (39.3%), older than 18 years, aged from 19 to 83 years (53.4 ± 1.26) with recurrent glioblastoma, Karnofsky Performance Score (KPS) 70% or higher. GBM patients were classified according to recent recommendation [9] to cover the diffuse astrocytic tumor with no molecular characterization. Also included was a group of patients (*n* = 55) with adenocarcinoma stage IV and brain metastasis from primary epithelial tumors (breast *n* = 19, 34.5%; lung *n* = 19, 34.5%; pancreas *n* = 2, 3.7%; colon *n* = 11, 20%; prostate *n* = 4, 7.3%) composed of 23 (41.8%) male and 32 (58.2%) female, older than 18 years with a mean age of 55.2 ± 1.92 years (range 29 to 89). A paired control group was also included of 130 healthy individuals of which 63 (51.5%) were male and 67 (48.5%) female, with a mean age of 55.6 ± 1.68 years (range from 18 to 95) without recent physical activity, and no clinical history of inflammatory, autoimmune or neurological processes, and living in the same geographic area as the patients. Pregnant or nursing women were considered ineligible.

### 4.2. Sample Collection and DNA Isolation

Blood sampling from patients was performed initially at the moment of protocol enrollment (S_0_, before ITN-POH treatment) and periodically during the inhalation therapy (S_1_, S_2_, S_n_), according to the availability of patients. cfDNA was extracted from freshly processed blood serum [28,41] with the QIAamp DNA Blood^®^ kit (Qiagen, Hilden, Germany) in compliance with manufacturer instructions. Quantification of the extracted DNA was performed by fluorimetry (Qubit^®^ Quanti-iT dsDNA BR Assay kit, Invitrogen, São Paulo, Brazil). DNA samples were stored at −20 °C until the moment of the use.

### 4.3. Drug Administration and Dose Escalation

POH was formulated for intranasal delivery and the preparation supplied by the University Pharmacy according to following patents: US Patent Application 20040087651 6 May 2004, and BR Application Number PI 0107262-5 17 December 2012. Perillyl alcohol (Sigma Chem. Co., St. Louis, MI, USA) 0.3% *v*/*v* (55 mg) was administered to all patients 4 times daily by intranasal (inhalation) delivery and the protocol consisted of an initial dose of 66.7 mg/dose (totaling 266.8 mg/daily) and then escalation to a 133.4 mg/dose (totaling 533.6 mg/daily).

### 4.4. Patient Evaluation

Patient response assessment was defined [42] and classified accordingly to the brain imaging (MRI) lesion as Complete Response: no detectable lesions during ITN-POH treatment; Partial Response: 30% reduction of largest diameter lesion; Stable Disease: images with no considerable variation in target lesion size; Progressive Disease: emergent new lesion and/or increment of the existing lesion. In addition, good responders were defined based on survival above 2 years of ITN-POH based therapy. Survival analysis was considered from the moment of patient inclusion at the clinical trial (S_0_) and 6 months was defined as the cut-off value, considering that all patients enrolled into the ITN-POH protocol were at a terminal stage, after failing all standard therapeutic options.

### 4.5. Statistical Analysis

Comparisons between three or more independent groups were performed by one-way ANOVA (followed by Kruskal–Wallis post-test). Comparison between two unmatched groups was performed by nonparametric unpaired *t*-test, followed by the Mann–Whitney test. Point-biserial Spearman correlation was chosen to evaluate the correlation between the variables serum circulating cfDNA concentration and patient survival. cfDNA diagnostic power was evaluated by receiver operating characteristic (ROC) curves and areas under the curves (AUC) were calculated. AUC values closer to the highest limit of 1 defined better test performance and AUC values closer to the lowest limit of 0.5 were related to the poorest outcome [43,44]. Statistically significant results were considered for a two-side p value of ≤0.05. GraphPad Prism 5 and SPSS 20.0 (IBM Inc., Chicago, IL, USA) were employed for the above-mentioned analyses.

## 5. Conclusions

The present data support previous reports describing a marked increase in circulating DNA concentrations in critically ill patients and highlight its relevance for disease prognosis and a favorable prognostic indicator for patients with brain tumor (GBM, brain metastasis) under POH-based therapy. Circulating cfDNA emerges as a noninvasive blood-based screening tool for early detection of tumor recurrence, as well as minimal residual disease and with the potential of monitoring the response to POH-based therapy.

## Supplementary Materials

Supplementary materials can be found at http://www.mdpi.com/1422-0067/19/6/1610/s1.

## Abbreviations

AUC	Area under the curve
BBB	Blood brain barrier
BM	Brain metastasis
cfDNA	Circulating cell-free DNA
CNA	Circulating nucleic acids
CSF	Cerebrospinal fluid
GBM	Glioblastoma
IDH	Isocitrate dehydrogenase
ITN	Intranasal
MGMT	O^6^-Methylguanine DNA methyltransferase
MRI	Magnetic resonance image
PET	Positron emission tomography
POH	Perillyl alcohol
ROC	Receiver operating characteristic
TMZ	Temozolomide
WHO	World Health Organization
