# Supplementary material for: Circulating Cell-Free DNA as a Prognostic and Molecular Marker for Patients with Brain Tumors under Perillyl Alcohol-Based Therapy
Source: Int J Mol Sci. 2018 May 30;19(6):1610. doi: 10.3390/ijms19061610 (PMC6032335; doi:10.3390/ijms19061610)
Supplement: Supplementary file 1 [file ijms-19-01610-s001.pdf]

Table S1. cfDNA serum levels between patients (GBM, BM) and controls (Ctrl).

cfDNA Serum Levels (ng/mL)				
	**N**	**Range**	**Median**	**Mean ± SE**
**GBM**	122	31,249 (101–31,350)	286	1237.50 ± 314.29
**BM**	55	9290 (40–9330)	588	1237.44 ± 255.21
**Control**	130	47 (38–85)	40	40.48 ± 0.52 *

* $p < 0.0001$.
